# Age-Related Differences in Clinical Outcomes of Patients with Pleural Empyema: A Retrospective Single-Center Study

**DOI:** 10.3390/geriatrics10040095

**Published:** 2025-07-18

**Authors:** Josef Yayan, Christian Biancosino

**Affiliations:** 1 Department of Internal Medicine, Division of Pulmonary, Allergy and Sleep Medicine, HELIOS Clinic Wuppertal, Witten/Herdecke University, Heusnerstr. 40, 42283 Wuppertal, Germany; 2 Department of Thoracic Surgery, HELIOS Clinic Wuppertal, Witten/Herdecke University, 42283 Witten, Germany

**Keywords:** pleural empyema, elderly, thoracotomy, VATS, surgical outcomes, age comparison

## Abstract

**Background**: Pleural empyema remains a serious clinical condition with high morbidity and mortality, especially in elderly patients. As life expectancy increases, a growing number of older individuals require surgical treatment. This retrospective single-center study investigated age-related differences in clinical presentation, perioperative features, and postoperative outcomes in patients undergoing surgery for pleural empyema. **Methods**: We conducted this retrospective study at Helios University Hospital Wuppertal, Witten Herdecke University in Germany, from December 2019 to May 2024. We stratified the patients into two age groups: <65 and ≥65 years. We compared baseline characteristics, American Society of Anesthesiologists (ASA) physical status classification, empyema stage, hospital stay, drainage duration, complication rates, and in-hospital mortality. **Results**: A total of 103 patients were included, of whom 43 (41.7%) were aged ≥ 65 years. Older patients had significantly higher ASA scores and presented with more advanced empyema stages. Hospital stay was significantly longer in this group. However, complication rates (60.0% vs. 44.9%; *p* = 0.25), drainage duration, ICU admissions (91.4% vs. 83.7%; *p* = 0.48), and in-hospital mortality (0% in both groups) did not differ significantly. **Conclusions**: Although older patients had higher perioperative risks, their surgical outcomes were similar to those of younger patients. Chronological age alone should not be a limiting factor for surgical treatment of pleural empyema. Surgical decisions should be based on clinical condition rather than chronological age.

## 1. Introduction

Pleural empyema is a serious infectious complication of the pleural space, most commonly arising from bacterial pneumonia, thoracic surgery, or chest trauma. Without timely intervention, it may lead to substantial morbidity and mortality due to persistent infection, respiratory failure, and systemic sepsis [1].

Surgical management remains the cornerstone of therapy in advanced stages (Stages II and III), especially when conservative measures and chest tube drainage fail [2]. Video-assisted thoracoscopic surgery (VATS) is increasingly used as a minimally invasive alternative to thoracotomy, offering benefits such as reduced postoperative pain, shorter hospital stays, and faster recovery, while maintaining efficacy in infection control and lung re-expansion [3,4].

Despite these advantages, the role of VATS in elderly patients remains controversial due to concerns about comorbidities and perioperative risks [5]. In addition, older patients with pleural empyema often present with advanced disease stages and live with frailty, having reduced physiological reserve, which may complicate surgical decision making [6]. However, the current literature on age-related surgical outcomes is limited and frequently inconsistent [7,8].

Given the ongoing demographic shift and the increasing incidence of empyema among elderly individuals, it is crucial to understand how age influences surgical outcomes [9]. This understanding is essential for informed risk assessment and individualized surgical planning.

The present retrospective study aimed to evaluate clinical characteristics, surgical approaches, and postoperative outcomes in patients treated surgically for pleural empyema at a tertiary care center. We compared outcomes between older (≥65 years) and younger (<65 years) adults, with a particular focus on surgical technique (VATS vs. thoracotomy), complication rates, and in-hospital course.

## 2. Materials and Methods

### 2.1. Study Design and Setting

We conducted this retrospective study at the Department of Thoracic Surgery at Helios University Hospital Wuppertal, a tertiary academic center affiliated with Witten/Herdecke University in Germany. The objective was to compare outcomes between older and younger adults undergoing surgery for pleural empyema.

### 2.2. Study Period and Patient Population

All adult patients who underwent surgical treatment for pleural empyema from December 2019 to May 2024 were screened for inclusion for their first episode of pleural empyema. Patients were eligible if they had a confirmed diagnosis of pleural empyema and had undergone either video-assisted thoracoscopic surgery (VATS) or open thoracotomy. We excluded patients who had undergone surgery for other indications or if key clinical data were missing. Participants were stratified by age into two groups: older adults (≥65 years) and younger adults (<65 years).

### 2.3. Data Collection

We retrospectively extracted data from electronic hospital records. We collected demographic data, including age and sex, as well as clinical characteristics, including smoking status, anticoagulant use, and American Society of Anesthesiologists (ASA) physical status classification, and the presence of comorbidities such as arterial hypertension, diabetes mellitus, coronary artery disease, chronic obstructive pulmonary disease, obesity, and chronic kidney disease. We recorded preoperative laboratory values, including hemoglobin, leukocyte count, creatinine, and C-reactive protein levels. We documented intraoperative parameters such as the laterality of the empyema, surgical duration, and microbiological findings. Postoperative outcomes included complications, ICU admission, in-hospital mortality, and length of hospital stay.

### 2.4. Surgical Management

Board-certified thoracic surgeons performed all procedures. The decision to undertake surgical intervention was based on clinical and radiological evaluation. The specific surgical approach—VATS or thoracotomy—was chosen based on imaging findings, intraoperative conditions, and the surgeon’s discretion. All patients received standardized perioperative management, including antibiotic therapy and postoperative chest tube drainage. ICU monitoring was guided by clinical status and procedural complexity. The operating surgeon chose between VATS and thoracotomy based on surgeon discretion, imaging findings, and the expected technical feasibility. The absence of a standardized selection protocol may have introduced selection bias. 

### 2.5. Statistical Analysis

We performed the statistical analysis using VassarStats (accessed on 1 May 2025, http://vassarstats.net), an online tool for statistical computation. Continuous variables were tested for normality and presented as mean with standard deviation. Between-group comparisons were conducted using the unpaired *t*-test for normally distributed variables or the Mann–Whitney U test for non-parametric data. We expressed categorical variables as absolute and relative frequencies and compared them using the chi-square or Fisher’s exact test, depending on sample size. Confidence intervals for proportions were calculated using the Wilson method. We considered a two-sided *p*-value < 0.05 statistically significant.

## 3. Results

### 3.1. Main Cohort Analysis

**Figure 1:**
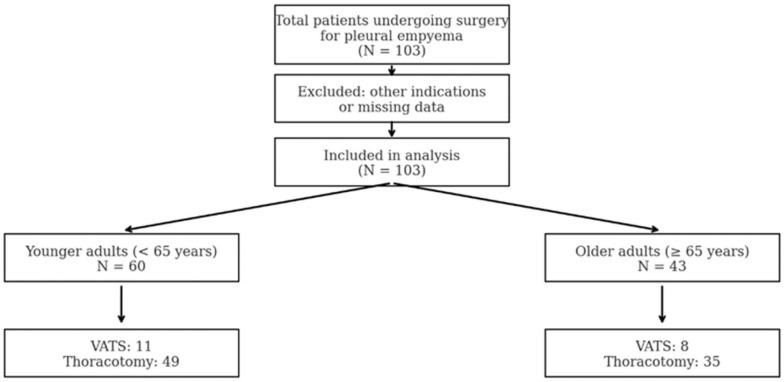
Patient flowchart illustrating cohort selection and surgical stratification. A total of 103 patients who underwent surgery for pleural empyema were included in the final analysis. We stratified patients by age into two groups: younger adults (<65 years) and older adults (≥65 years). Within each age group, the surgical approach was further categorized into video-assisted thoracoscopic surgery (VATS) or open thoracotomy.

A total of 103 patients underwent surgical treatment for pleural empyema between 1 December 2019 and 31 May 2024, at the Department of Thoracic Surgery, Helios University Hospital Wuppertal, affiliated with Witten/Herdecke University. Of these, 43 patients (41.7%) were classified as older adults (≥65 years), and 60 patients (58.3%) as younger adults (<65 years). Table 1 summarizes the baseline characteristics of both age groups. Comparisons revealed notable differences in age-related parameters and perioperative risk profiles. The patient flowchart is shown in Figure 1.

Older adults were more likely to be on blood-thinning medication compared to younger adults (25.7%, 95% CI: 14.2–42.1% vs. 8.2%, 95% CI: 3.2–19.2%; *p* = 0.04). ASA I classification was only observed in younger adults (10.2%, 95% CI: 4.4–21.8%), whereas it was absent in older patients (0.0%, 95% CI: 0.0–9.9%). ASA III classification tended to be more common in the older group (71.4%, 95% CI: 54.9–83.7% vs. 61.2%, 95% CI: 47.2–73.6%; *p* = 0.46). ASA II distribution was comparable between groups.

The prevalence of arterial hypertension was similar (28.6 %, 95 % CI: 16.3–45.1 % in older adults vs. 32.7%, 95% CI: 21.2–46.6% in younger adults; *p* = 0.87), as were diabetes mellitus (11.4%, 95% CI: 4.5–26.0% vs. 12.2%, 95% CI: 5.7–24.2%), obesity (5.7%, 95% CI: 1.0–19.1% vs. 4.1%, 95% CI: 0.7–16.7%), COPD (11.4%, 95% CI: 4.5–26.0% vs. 12.2%, 95% CI: 5.7–24.2%), and coronary heart disease (2.9%, 95% CI: 0.5–14.5% vs. 8.2%, 95% CI: 3.2–19.2%), with no statistically significant differences.

We found no significant differences in sex distribution (male: 77.1%, 95% CI: 60.0–88.5% vs. 69.4%, 95% CI: 55.3–80.6%; *p* = 0.59) or current or past smoking status (40.0%, 95% CI: 25.2–56.7% vs. 42.9%, 95% CI: 30.0–56.7%; *p* = 0.97). The distribution of right- and left-sided empyema, as well as intraoperative detection of microorganisms, was comparable.

**Figure 2:**
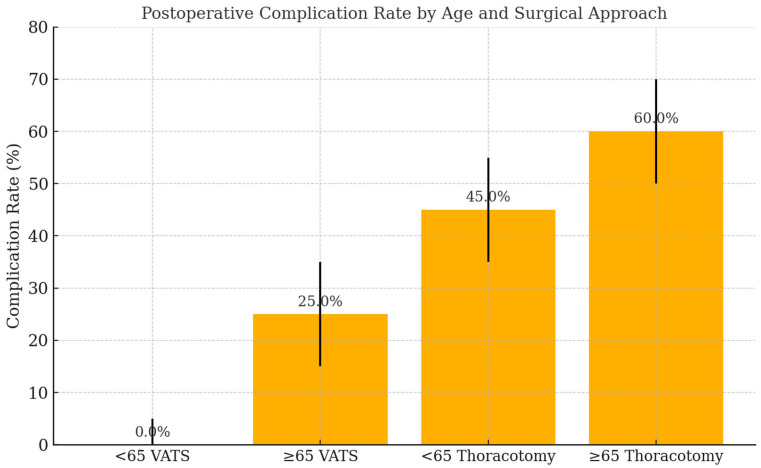
Postoperative complication rates stratified by age group and surgical approach. Younger (<65 years) and older (≥65 years) patients were further categorized by surgical technique: video-assisted thoracoscopic surgery (VATS) or thoracotomy. Complication rates were numerically higher in elderly patients undergoing thoracotomy, although not statistically significant. The vertical black ticks represent the error bars, indicating the variability (standard error or confidence interval) in the complication rates for each group.

Postoperative complications occurred more frequently in older adults (60.0%, 95% CI: 43.3–74.8%) than in younger patients (44.9%, 95% CI: 31.8–58.7%), although this difference was not statistically significant (*p* = 0.25). Postoperative complications in elderly patients included both major and minor events (Figure 2). Major complications included respiratory failure, prolonged air leak, and re-intervention, though numbers were limited. ICU admission was more frequent in the older group (91.4%, 95% CI: 77.6–97.0%) compared to the younger group (83.7%, 95% CI: 70.9–91.8%; *p* = 0.48). The need for blood transfusion was identical in both groups (28.6%, 95% CI: 16.3–45.1%). No in-hospital mortality occurred in either group (Table 2).

Laboratory findings including hemoglobin (11.4 ± 1.8 vs. 11.1 ± 1.8 g/dL; *p* = 0.55) (Table 1 and Table 2), leukocyte counts (12,920 ± 6432 vs. 13,417 ± 5096/μL; *p* = 0.37), creatinine (1.0 ± 0.4 vs. 1.0 ± 1.2 mg/dL; *p* = 0.054), and CRP (10.9 ± 7.8 vs. 11.4 ± 7.8 mg/dL; *p* = 0.25) did not differ significantly. The duration of surgery was comparable between groups (104.7 ± 60.2 vs. 117.0 ± 37.6 min; *p* = 0.89).

### 3.2. Subgroup Analysis by Surgical Approach

A subgroup analysis of the VATS cohort revealed similar trends: significantly lower hemoglobin values in older patients (*p* = 0.017), higher rates of ASA III classification, and numerically higher complication and ICU rates, though without statistical significance. Table 1 shows the subgroup analysis for VATS patients; Table 3 presents the subgroup analysis for thoracotomy patients.

## 4. Discussion

This retrospective study compared clinical characteristics, surgical management, and postoperative outcomes between older (≥65 years) and younger (<65 years) adults undergoing surgery for pleural empyema at a tertiary care center. Despite the higher burden of comorbidities among older patients, our results demonstrate that surgical outcomes—including complication rates, ICU admissions, and in-hospital mortality—were largely comparable between age groups.

Older patients were significantly more likely to receive anticoagulant therapy, which likely reflects a higher prevalence of cardiovascular conditions such as atrial fibrillation and coronary artery disease in this population [10]. Importantly, despite the increased use of blood-thinning medications, no rise in bleeding complications or transfusion requirements was observed in elderly patients, which is consistent with previous studies [11].

ASA classification, an established indicator of perioperative risk, showed clear age-related differences. ASA III status was more common among older adults, while ASA I was exclusively observed in the younger group. This finding is consistent with the expected decline in physiological reserve with aging [12]. However, despite these higher ASA scores, there were no significant differences in surgical duration, ICU admission, or complication rates, suggesting that ASA classification alone does not reliably predict adverse surgical outcomes in pleural empyema as it does not assess frailty, which represents a distinct and important dimension of preoperative risk [13]. A key limitation of our study is the absence of a formal frailty assessment. While age and ASA classification reflect certain aspects of risk, neither fully captures functional or physiological reserve. Future studies should incorporate validated frailty scores to improve risk stratification and assess the generalizability of surgical outcomes in older adults.

Although complication rates were numerically higher in older patients (60.0% vs. 44.9%), this difference did not reach statistical significance. Moreover, ICU admission was more frequent in elderly patients, which may reflect a more cautious postoperative monitoring approach rather than actual clinical deterioration. Similar observations have been reported in thoracic surgery literature, where advanced age was not independently associated with worse postoperative outcomes when perioperative care was appropriately managed [14,15].

Of note, no in-hospital mortality occurred in either group, underlining the safety of surgical treatment for pleural empyema across age groups. The low mortality rate in our cohort may reflect institutional standards of care, early surgical intervention, and multidisciplinary collaboration, including close perioperative monitoring and infection management.

Laboratory parameters, including inflammatory markers and renal function, did not significantly differ between age groups. Although older patients had significantly lower preoperative hemoglobin levels, this did not translate into an increased need for transfusion or adverse outcomes. These findings suggest that modest hematological variations in elderly patients may be tolerated without clinical consequence.

Subgroup analysis of the VATS cohort revealed similar trends. While older adults undergoing VATS had lower hemoglobin levels and slightly higher complications and ICU admission rates, these differences were not statistically significant. Despite common concerns about frailty and reduced cardiopulmonary reserve, our findings suggest that VATS is a feasible and safe option in elderly patients when carefully selected [16]. Reduced cardiopulmonary reserve in elderly patients—often due to prevalent COPD and cardiovascular disease—may increase the risk of postoperative respiratory complications. 

Taken together, our findings show that elderly patients undergoing the same surgical procedures as younger adults achieved comparable in-hospital outcomes. Therefore, chronological age alone should not be considered a contraindication for surgical treatment of pleural empyema.

## 5. Limitations

This study has several limitations. First, it was a retrospective, single-center analysis, which limits external validity and generalizability. Second, the sample size was relatively small—particularly in the subgroup undergoing VATS—which may have reduced the statistical power to detect subtle but clinically relevant differences in outcomes such as complications or ICU admissions.

Third, treatment allocation (VATS vs. thoracotomy) was not standardized or randomized, but rather based on surgeon discretion and clinical judgment. This introduces a potential selection bias that may have influenced perioperative outcomes.

Fourth, although baseline characteristics such as ASA classification and hemoglobin levels differed between groups, these variables were not controlled for in multivariable analysis. This may confound the interpretation of age-related outcome differences.

Fifth, we focused solely on in-hospital outcomes. Long-term data on recurrence, pulmonary function, functional independence, and health-related quality of life were not available. These are important parameters for evaluating postoperative recovery and quality of care in elderly surgical patients and should be investigated in future prospective studies. Furthermore, we were unable to include validated frailty assessments or objective lung function parameters such as spirometry or blood gas analysis, which may have provided additional insights into surgical risk profiles.

A key limitation of our study is the absence of formal frailty assessment. Although ASA classification reflects comorbidity burden, it does not capture functional or physiological reserve. Future studies should incorporate validated frailty scores to better characterize surgical risk in elderly patients.

## 6. Conclusions

In this retrospective study of 103 patients undergoing surgery for pleural empyema, older adults (≥65 years) exhibited more comorbidities and higher ASA classifications than younger adults but achieved comparable in-hospital outcomes. We observed no significant differences in complication rates, ICU admissions, or perioperative mortality between age groups. Notably, no in-hospital deaths occurred in either group.

These findings suggest that advanced age alone should not be viewed as a contraindication for surgical treatment of pleural empyema. With appropriate perioperative evaluation and management, both thoracotomy and minimally invasive approaches such as VATS can be safely performed in elderly patients.

Future prospective, multicenter studies are needed to confirm these findings and to evaluate long-term outcomes, including pulmonary function, recurrence, and postoperative quality of life.

## Figures and Tables

**Table 1:** Subgroup analysis of patients undergoing video-assisted thoracoscopic surgery (VATS), stratified by age group. The demographic, clinical, and perioperative characteristics of patients who underwent VATS for pleural empyema, divided into those aged < 65 years and those ≥ 65 years, are shown.

Variable	Older Adults (≥65 Years) N = 8 (%)	Younger Adults (<65 Years) N = 11 (%)	*p* Value
Male	6 (75.0)	6 (54.5)	0.63
Female	2 (25.0)	5 (45.5)	0.63
Age (mean ± SD)	77.9 ± 8.0	41.3 ± 13.2	**<0.001**
Nicotine	3 (37.5)	4 (36.4)	1.0
Blood thinning	3 (37.5)	1 (9.1)	0.26
ASA I	0	3 (27.3)	0.23
ASA II	0	5 (45.5)	**0.045**
ASA III	7(87.5)	4 (36.4)	0.059
Complication	2 (25.0)	0	1.0
Hemoglobin (mean ± SD) (g/dL)	11.1 ± 1.2	12.6 ± 1.3	**0.017**
Intensive Care Unit (ICU)	4 (50.0)	7 (63.6)	0.66

**Abbreviations:** ASA, American Society of Anesthesiologists physical status classification; SD, Standard Deviation. **Note:** significant *p* values are in bold.

**Table 2:** This table presents the distribution of postoperative complications among patients undergoing surgery for pleural empyema, stratified by age group (<65 years vs. ≥65 years) and surgical approach (VATS vs. thoracotomy). Percentages refer to the proportion of patients within each subgroup who experienced the respective complication. A single patient may have had more than one complication. Statistical comparisons between groups were performed using the chi-square test ** or Fisher’s exact test *, as appropriate.

Complication	<65 Years (n = 60)	≥65 Years (n = 43)	VATS (n = 19)	Thoracotomy (n = 84)	*p* Values
Pneumonia	1 (1.7%)	5 (11.6%)	0	6 (7.1%)	0.0797 *
Prolonged air leak	4 (6.7%)	5 (11.6%)	1 (5.3%)	8 (9.5%)	0.4853 *
Respiratory failure	3 (5.0%)	4 (9.3%)	0	7 (8.3%)	0.4474 *
Re-intervention required	1 (1.7%)	2 (4.7%)	0	3 (3.6%)	0.5696 *
Surgical site infection	2 (3.3%)	1 (2.3%)	0	3 (3.6%)	1.0 *
≥1 complication	27 (44.9%)	26 (60.0%)	2 (10.5%)	45 (53.6%)	0.1774 **

**Table 3:** Subgroup analysis of patients undergoing thoracotomy, stratified by age group. The demographic, clinical, and perioperative characteristics of patients who underwent thoracotomy for pleural empyema, divided into those aged < 65 years and those ≥ 65 years, are shown.

Variable	Older Adults (≥65 Years) N = 35 (%)	Younger Adults (<65 Years) N = 49 (%)	*p* Value
Male	27 (77.1)	34 (69.4)	0.59
Female	8 (22.9)	15 (30.6)	0.59
Age (mean ± SD)	74.3 ± 6.2	49.5 ± 11.5	**<0.001**
Nicotine	14 (40.0)	21 (42.9)	0.97
Blood thinning	9 (25.7)	4 (8.2)	0.04
ASA I	0	5 (10.2)	0.07
ASA II	10 (28.6)	13 (26.5)	1.0
ASA III	25 (71.4)	30 (61.2)	0.46
Complication	21 (60.0)	22 (44.9)	0.25
Death	0	0	1.0
Pneumonia	5 (14.3)	1 (2.0)	0.08
Hemoglobin (mean ± SD) (g/dL)	11.4 ± 1.8	11.1 ± 1.8	0.55
Intensive Care Unit (ICU)	32 (91.4)	41 (83.7)	0.48

**Abbreviations:** ASA, American Society of Anesthesiologists physical status classification; SD, Standard Deviation. **Note:** significant *p* values are in bold.

## Data Availability

All data generated or analyzed during this study are included in this published article.
